# Report on von Willebrand Disease in Malaysia

**DOI:** 10.3889/oamjms.2016.030

**Published:** 2016-02-29

**Authors:** Mercy Halleluyah Periayah, Ahmad Sukari Halim, Arman Zaharil Mat Saad, Nik Soriani Yaacob, Faraizah Abdul Karim

**Affiliations:** 1 *Reconstructive Sciences Unit, School of Medical Sciences, Universiti Sains Malaysia, 16150 Kubang Kerian, Kelantan, Malaysia*; 2 *Department of Chemical Pathology, School of Medical Sciences, Universiti Sains Malaysia, 16150 Kubang Kerian, Kelantan, Malaysia*; 3 *Hemophilia Clinic, National Blood Centre (Pusat Darah Negara), Jalan Tun Razak, 50400, Wilayah Persekutuan, Kuala Lumpur, Malaysia*

**Keywords:** von Willebrand Disease, Malaysian report, Sociodemographic details, Laboratory profiles, Year 2011-2013

## Abstract

**BACKGROUND:**

Von Willebrand disease (vWD) is an inherited hemostatic disorder that affects the hemostasis pathway. The worldwide prevalence of vWD is estimated to be 1% of the general population but only 0.002% in Malaysia.

**AIM:**

Our present paper has been written to disclose the statistical counts on the number of vWD cases reported from 2011 to 2013.

**MATERIAL AND METHODS:**

This article is based on sociodemographic data, diagnoses and laboratory findings of vWD in Malaysia. A total of 92 patients were reported to have vWD in Malaysia from 2011 to 2013.

**RESULTS:**

Sociodemographic-analysis revealed that 60% were females, 63% were of the Malay ethnicity, 41.3% were in the 19-44 year old age group and 15.2% were from Sabah, with the East region having the highest registered number of vWD cases. In Malaysia, most patients are predominately affected by vWD type 1 (77.2%). Factor 8, von Willebrand factor: Antigen and vWF: Collagen-Binding was the strongest determinants in the laboratory profiles of vWD.

**CONCLUSION:**

This report has been done with great interest to provide an immense contribution from Malaysia, by revealing the statistical counts on vWD from 2011-2013.

## Introduction

Malaysia is one of the wealthiest and most developed countries in Southeast Asia with widespread and systematic healthcare system. The healthcare sector in Malaysia is primarily operated by the Ministry of Health Malaysia (MOH), established under Malaysian government [1]. In reviewing the Malaysian Health Profiles, coronary heart disease, stroke and respiratory illness are the leading causes of death, as recorded by the World Health Organization (WHO) in 2011, among 50 major etiologies of deaths [2]. Hospitals in Malaysia are very much dedicated in providing the most current advanced interventions to patients to save their lives from the deadliest diseases and to promote quality treatments. Hereditary hematological disorders affect the blood, and the diagnosis of such cases is more complicated due to the involvement of genes in the deoxyribonucleic acid (DNA) that interrupt the working mechanisms of blood clotting factors. Based on the recent update from the National Blood Centre (PDN) of Malaysia, there are only a few men and women suffering from these diseases, leading to the conclusion that the impact of these diseases is relatively low among Malaysians. PDN was officially built under public sector to provide expert medical services and a foundation for research in the field of hematology. PDN has also received accreditation from National Association of Testing Authorities (NATA), Australia for ISO/IEC 17025 and specialization in 17 different fields related to blood-associated diagnoses, resulting in quality medical outputs [3]. There are a few inherited hemostatic disorder cases reported by PDN repetitively every year, including Hemophilia, Von Willebrand disease (vWD), Bernard-Soulier Syndrome, adenosine diphosphate (ADP) receptor defect, Glanzmann Thrombasthenia and Factors deficiencies. PDN reports reveal that Hemophilia and vWD are the two major hemostatic disorders recorded at a high frequency level nationwide.

A mutation in the von willebrand factor (vWF) gene is the main cause of vWD. The vWF gene supplies crucial signals to form vWF blood-clotting proteins, which trigger the formation of blood clots. vWF acts as a sticky glue to hold the blood clots together in preventing excessive blood loss. At the same time, abnormal blood clotting causes prolonged bleeding episodes in vWD. Although the public attention towards global health has grown quickly over the past half century, the understanding and awareness of Malaysians towards hereditary hemostatic diseases are still considered to be poor in comparison with other ASEAN countries.

More needs to be done to coordinate Malaysians in order to improve the consciousness about these inherited hemostatic disorders. A total of five hundred fifty-four cases of vWD were reported in the PDN hemorrhagic disorders registry from 1979 to 2013. Five hundred seventy-two vWD events were registered in Malaysia as of 2013. In Malaysia, many campaigns, colloquia and conferences have been organized, but to date, there are no updates or published evidence surveying the statistics of vWD over the last 3 years.

Our present paper aims to disclose the statistic counts or percentages on vWD in Malaysia and to provide the report for the past 3 years, from 2011 to 2013, regarding the age, gender, race, region and diagnosis (approved by MOH). This report is a contribution from Malaysia with the clinical description of vWD to the countries all over the world.

## Material and Methods

Prior to commencing the study, ethical clearance was obtained from the Medical Research & Ethics Committee from National Medical Research Registry (NMRR). This study has been registered with NMRR and the research identification number is NMRR-13-873-17276. The vWD patient entries in the PDN registry are from various hospitals across Malaysia. Reports on the statistics were prepared between 2011 and 2013 and included selected demographic profiles (age, gender, ethnicity, state, region) and other data including vWD classification, year of diagnosis (2011-2013) and laboratory findings includes [Factor 8 (FVIII), vWF: Antigen (vWF: Ag) and vWF: Collagen-Binding (vWF: CB)]. In our analyses, we categorized patients into 5 different age categories: (0-4), (5-13), (14-18), (19-44) and (45 and above) years old.

Ethnicities were classified based on the major populations of Malaysia, which are Malay, Chinese, and Indian, followed by Kadazan and Dusun, that distinguished in the ‘others’ category. Patients were also identified by their native states: eleven states (Perlis, Kedah, Pulau Pinang, Perak, Selangor, Negeri Sembilan, Melaka, Johor, Pahang, Terengganu, Kelantan) and 3 federal states (Wilayah Persekutuan, Sabah, Sarawak). All of the above listed states are divided into 2 separate regions, Peninsular Malaysia and East Malaysia.

The classifications of vWD diagnosis were split into 3 different categories, listed as type 1, 2 and 3. Laboratory findings expressed in percentages (FVIII, vWF: Ag and vWF: CB) were assigned into 5 distinguished categories, below 10, 10-30, 31-60, 61-100 and above 100. The data points were presented in percentages by using cross-tabulation between the demographic data. The independent t-test was applied to compare the mean and standard deviation (SD) of the diagnosis type and the laboratory findings. The statistical analysis was employed using SPSS version 18.0 software. Detailed data on the number of vWD incidences in Malaysia were solely provided by PDN.

## Results

### Sociodemographic characteristics

**Figure 1:**
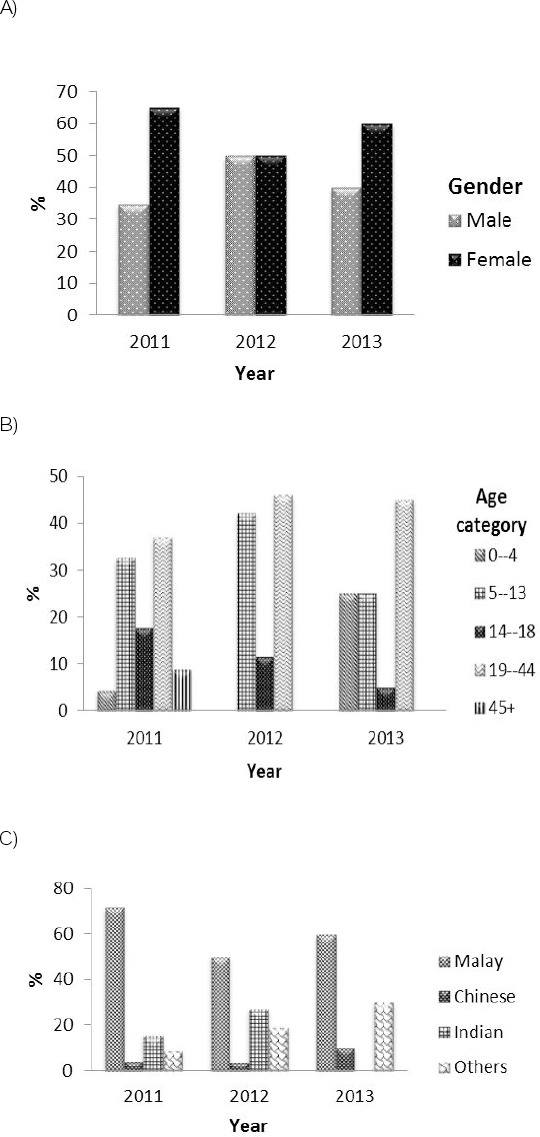
*Percentages of gender, age category and ethnic distribution of vWD patients (n = 92) diagnosed in the PDN from 2011 to 2013, respectively. The analyses were expressed in percentages using descriptive statistics*.

Ninty-two patients between 1 and 52 years old who presented with vWD were recruited and diagnosed by PDN within 2011 to 2013. Out of 92 patients, 40% were males and 60% were females. The majority of patients in this 3 year period were recorded in 2011 with 46 patients (35% of males and 65% of females). In 2012, both genders registered in equivalent percentages. In 2013, the lowest number of cases was registered, with a total of 20 patients at a 2:3 ratio of males to females (Fig. 1A).

Cases of vWD were categorized into 6 different age classifications. Patients between 19-44 years old were most commonly diagnosed within these 3 years (41.3%), followed by patients 5-13 years old (33.7%). Only 4.4% of registered patients were above 45 years old (Fig. 1B). The ethnicity distribution of the 92 total patients presenting with vWD to PDN in 2011 to 2013 is depicted in Fig. 1C. The majority of patients (63%) were the main ethnicity of Malaysia, Malay. Ethnicities falling under the categories of ‘others,’ Indian and Chinese represented 16.3%, 15.2% and 5.5% of the total, respectively.

### States and Region

**Figure 2:**
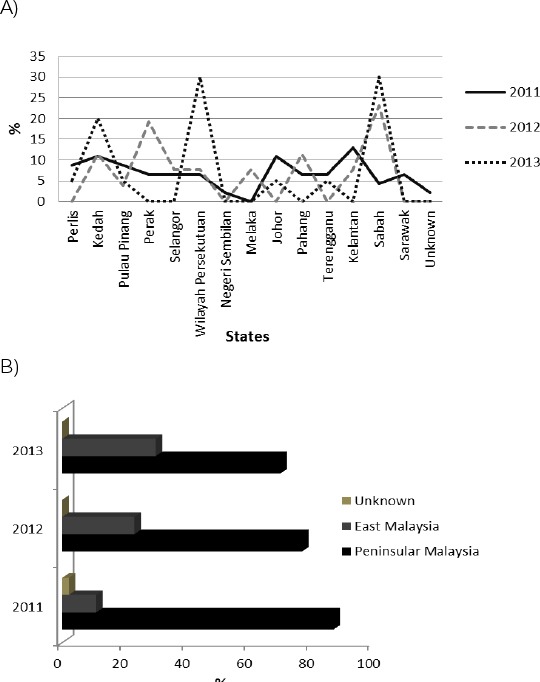
*Frequency of vWD patient native states, federal territories and regions, depicted in percentages. The figures illustrate the data of the patients diagnosed in the PDN for 3 consecutive years from 2011 to 2013; n=92*.

The majority of patients were from Sabah in the East Malaysia region, representing an overall percentage of 15.2%, followed by Kedah in 13.0% of patients, and Wilayah Persekutuan in 12.0%. Noticeably, only 1.1% of patients diagnosed in PDN were from the Negeri Sembilan state in the 3 consecutive years studied. Similarly, only 1 patient was considered to be of unknown origin (Fig. 2A). Eleven different states and 1 federal state comprise Peninsular Malaysia. The east coast region only consists of 2 distinct federal territories, Sabah and Sarawak. As for the overall analysis, most patients were from Peninsular Malaysia [74 (80.4%)], with only 17 cases from the East Coast region (18.5%). Although the total area of East Coast region represents approximately 61% of the total land area of Malaysia, the highest sum of vWD cases were recorded in the peninsular region, as it contains the most states (12) and is comprised of 22 million residents Fig. 2B.

### Classifications of vWD; Laboratory findings; Year of diagnosis

**Figure 3:**
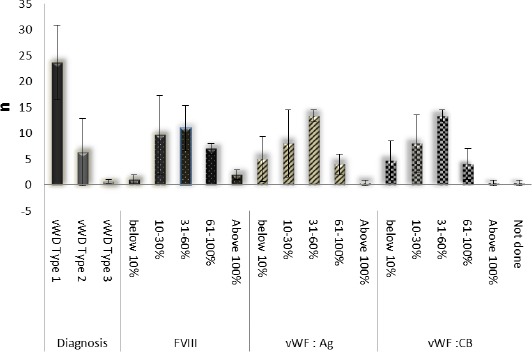
*Classifications of vWD as Type 1, 2, and 3, and the laboratory findings of (FVIII, vWF: Ag, vWF: CB) based on the year of diagnosis (2011 to 2013). The outcome of the laboratory profiles classified into 5 different grades, below 10, 10-30, 31-60, 61-100, and above 100, is shown in percentages. The data were analyzed by the Independent t-test and are presented in means (SD); n=92*.

The laboratory findings of each patient were depicted as percentages and the outcomes of each category were expressed as the mean (SD). It has been revealed that most patients are predominantly affected by vWD type 1 (77.2%) of patients throughout the 3 sequential years (2011-2013), with a mean (SD) value of 24 (7.23), as outlined in Fig. 3. The associations between types of vWD were strong factors influencing the laboratory findings. The highest prevalence recorded for all 3 distinct laboratory findings by groups of vWD patients was in the 31-60% group, with mean (SD) values as follows; FVIII: 11 (4.41), vWF:Ag: 13 (1.16) and vWF:CB: 13 (1.16). The next highest category was 10-30%, with mean (SD) values as follows; FVIII: 10 (7.64), vWF: Ag: 8 (6.56), vWF: CB: 8 (5.57).

## Discussion

The term vWD was coined by Finnish physician Eric Adolf von Willebrand in 1926. vWD is an inherited hemostatic disorder that affects the hemostasis pathway [1]. According to the recent annual global survey by the World Federation of Hemophilia updated in 2010, only 0.002% of vWD cases were reported among the population of 28 million Malaysians. Apparently, the Malaysian Statistical Department website has posted that the population of Malaysia has increased to 30 million, and that the counts now represent 0.043% of the world population. The worldwide prevalence of vWD is estimated to be 1%, and based on our recent analysis, vWD cases are estimated to be found in 0.002% of the total population [4]. PDN has diagnosed a total number of 554 cases of vWD from early 1979 to 2013. Five hundred seventy-two vWD cases have been officially diagnosed and reported in the Malaysian Health registry, which comprises 554 cases in the PDN, 6 cases from University hospitals and 12 cases diagnosed in state general hospitals.

vWD arises from a defect due to abnormal vWF, which disrupts platelet aggregation mechanisms. vWF, formed between endothelial cells, is a large multimeric protein that helps platelets adhere and aggregate in order to facilitate hemostasis [5]. Ninety-two cases of vWD were recorded in patients between 1 to 52 years old from 2011 to 2013. The numbers of female patients recorded was 20% higher than the number of males. We have categorized the age of the patients into 6 distinct categories. This age classification was adapted from a previous report on Hemophilia in Singapore described by Kwa *et al.*, [6]. The analysis showed that patients in the age category of 19-44 years old showed the highest number of registered vWD cases, with a total of 38.

Although the Malaysian population comprises multiple ethnicities, the majority of the Malaysian population was of the native ethnicity, often referred to as the Malays or Bumiputeras. The prevalence of vWD cases was highest in Malays (63%) and lowest in Chinese (5.5%). The ratio of the overall number of events based on ethnicity for both sexes standardized by age and relative to Malay are as follows: (Chinese: Malay; 1:11), (Indian: Malay; 1: 4.1) and (Others: Malay; 1:3.9). The Indian population lived in Malaysia are comparatively lower than the Chinese population in Malaysia, with only 7.1% Indians in the total Malaysian population. Indian descendant are recorded as the second most common ethnicity affected by vWD. Malaysia is well-known as a multiracial country consisting of 3 major ethnicities, Malay, Chinese, Indian and others. The ethnicities that fall under the ‘others’ category are Kadazan, Dusun, Iban, Minangkabau, Murut, Sikh, Bajau, Baba-Nyonya, Orang Asli, Dayak, Orang Ulu and Bidayuh. Based on our analysis, we found that of the ‘others’ category, only patients of the Kadazan and Dusun ethnicities has been diagnosed with vWD. Geographically, Malaysia is divided into 2 regions, Peninsular Malaysia and East Malaysia. Within these regions, Malaysia is divided again into 11 states and 3 federal territories. The majority of the vWD patients originated from Sabah, and this statement could be significantly correlated with the number of patients detected based on ethnicity. In Malaysia, the majority of the Kadazan and Dusun ethnicities originated from Sabah. Because other ethnic groups mostly lived in that state, together with the 3 major ethnic groups, this could explain why Sabah was reported to have the highest number of vWD cases.

vWD is an inherited disorder, so most of the patients diagnosed in PDN were from the same family. We also understand that patients who categorized under their native places were subject to change locations, because many people tend to migrate due to personal needs or for work purposes. Classification of vWD, including Type 1, Type 2A, 2B, 2 M, 2N, 3 and pseudo-type, based on the hereditary pattern, mechanism, prevalence in the general population, frequency of occurrence among vWD patients and bleeding tendency [7,8]. In Malaysia, among the majority of the 92 reported vWD patients, we encountered that the 97.9% of patients reported as having type 1 or 2 vWD, falling into the mild to moderate category. Subsequently, vWD type 3 occurred in only 2.1% of cases. The clinical assessment of a person who has identified as a vWD patient should be classified and diagnosed according to their family history and laboratory profile outcomes. In medical practice, laboratory diagnosis could vary according to the patient’s body and immune responses. Therefore, the laboratory profiles for vWD patients could be markedly different from the assumed and expected outcomes. These profiles are extremely dependent on the vWD classification, family history and response towards provided treatment [9-14].

In PDN, vWD patients were typically classified based on the 3 important laboratory findings: the assessment of FVIII, vWF: Ag and vWF: CB. These laboratory observations were categorized into 5 different measurement levels, as mentioned above. Patients with laboratory measurements below 10% are considered to have extremely low levels and are categorized as cases that are very severe and rare, while measurements between 10-30%, 31-60%, or 61-100% are assigned as severe to mild cases. Subsequently, levels above 100% are considered normal as type 1 vWD. In the 3 years studied, no mortality due to vWD was reported due to prolonged bleed. As for the overall view, no statistically significances were observed between gender, year of diagnosis or type of diagnosis.

It was reported by Mohsin *et al.*, that many cases of vWD remain undiagnosed due to various types of clinical manifestations and perplexing laboratory analysis. Patients who have insufficient amounts of vWF, less than 20 IU/dL, are most likely to have vWF gene mutations with significant bleeding tendencies [11, 15]. There are actually two different types of bleeding: mucocutaneous bleeding and traumatic bleeding. Mucocutaneous bleeding normally affects the mucous membranes, which are the delicate tissues lining the body passages such as the nose, mouth, uterus, vagina, stomach and intestines. This type of hemorrhages frequently occurs upon epistaxis, gingival bleeding, menorrhagia, gastrointestinal bleeding and superficial ecchymosis (bruising). In contrast, traumatic bleeding occurs due to surgery, childbirth, larger injuries and tooth extractions.

There are two different types of treatment options that can be issued to vWD patients, desmopressin (DDAVP) and transfusion therapy, depending on the degree of severity and bleeding tendency. DDAVP is the standard form of therapy for patients experiencing Type 1 and 2A vWD. This drug mainly elevates the release of vWF and FVIII from endothelial cells and, in addition, increases the levels of vWF and FVIII:C by 3 to 5-fold. DDAVP can be administered intranasal and intravenously. In contrast, DDAVP is resistant and contradictory in patients with Type 2B vWD. This is due to their risk of developing thrombocytopenia. DDAVP has also been discovered to be an ineffective drug for Type 2N, 2 M and 3 vWD. Although antifibrinolytic (Tranexamic acid, epsilon-aminocaproic acid) and plasma-based drug substances have not been widely been used to treat Type 1 vWD, these compounds are the mainstay to treat patients with type 2 and 3 vWD.

Special cases that present with alloantibodies can be treated with recombinant FVIII and recombinant activated FVIII. Women undergoing heavy menstrual bleeding will usually be treated with oral contraceptive medications containing estrogen, which are more likely to be very effective in reducing the time frame of menorrhagia. For pregnant patients, they will be monitored accordingly to avoid excessive bleeding, especially during the initial postnatal weeks. Human-derived medium purity Factor VIII concentrates, which contain vWF, are available for patients undergoing surgical interventions with vWD complications. Humate P, Alphanate and Koate HP are also commercially available for prophylaxis of vWD. Platelet concentrates can be transfused for vWD patients categorized as having pseudo-type vWD, and blood transfusions can be given to prevent anemic and hypotensive cases among vWD patients [8, 11]. Many vWD patients in Malaysia only experience a mild form of this disease, which normally does not cause any serious hemorrhage. Patients identified under the severe category should seek emergency treatment to cease bleeding before it could become life-threatening. On the other hand, topical hemostatic agents such as thrombin, chitosan-derivatives, floseal, lyostypt have also been employed extensively to stop the hemorrhage from any injuries.

In conclusion, vWD is the second most common hemostatic disorder reported to occur in Malaysia, followed by Hemophilia. Our analysis has demonstrated important temporal changes in the demographics and the laboratory findings of vWD cases diagnosed in the PDN in Malaysia from 2011 to 2013. A total of 92 vWD cases were registered in Malaysia from 2011 to 2013. In Malaysia, most patients are affected by type 1 vWD. In reviewing the patient’s sociodemographic characteristics, we noticed that most vWD cases occurred among females, patients of the Malay ethnicity and in the 19-44 year old age group. The East region and Sabah state registered the highest number of vWD cases. This report has been done with great interest to provide an immense contribution from Malaysia, by revealing the statistical review on vWD. Based on this report, it seems apparent that the occurrence of vWD could be increasing along with the population of Malaysia; therefore, it is important to address the clinical care treatments for vWD patients before the disease becomes life-threatening.
